# Immunomodulatory roles of butyrate in asthma: mechanisms and therapeutic potentials

**DOI:** 10.3389/fimmu.2025.1639606

**Published:** 2025-08-11

**Authors:** Chao Liu, Zhu Zeng, Mei Chen, Yuwei Fan, Qingsong Huang, Jianying Wu

**Affiliations:** ^1^ Department of Integrated Traditional Chinese and Western Clinical Medicine, School of Clinical Medicine, Chengdu University of Traditional Chinese Medicine, Chengdu, China; ^2^ Department of Respiratory Medicine, Hospital of Chengdu University of Traditional Chinese Medicine, Chengdu, China; ^3^ School of Medical and Life Sciences, Chengdu University of Traditional Chinese Medicine, Chengdu, China; ^4^ Department of Gastroenterology, Hospital of Chengdu University of Traditional Chinese Medicine, Chengdu, China

**Keywords:** butyrate, type 2 innate lymphoid cells, histone deacetylase, short chain fatty acids, immunity

## Abstract

Asthma, a chronic airway inflammatory disease driven by complex immune dysregulation, still remains a global health challenge despite its advances in biologic therapies. Butyrate, a major short-chain fatty acids (SCFAs) produced by intestinal microorganisms in the fermentation of dietary fiber, has recently garnered considerable attention for its multifaceted roles in maintaining immune homeostasis and modulating airway inflammation. This review summarizes the molecular mechanisms and recent advances by which butyrate alleviates asthmatic inflammation, including suppression of excessive activation of type 2 innate lymphoid cells (ILC2s) and T helper 2 (Th2) cells, inhibition of mast cells (MCs) degranulation, epigenetic modulation, regulation of receptor-mediated signaling pathways, and interactions along the gut–lung axis. We integrate current knowledge of butyrate’s multidimensional immunoregulatory network in asthma and propose a dual approach—via microbiota-based interventions and targeted modulation of the immune microenvironment—to potentially overcome the limitations of conventional corticosteroid therapies. Despite its promising prospects, its clinical translation still faces many challenges, especially in airway specific delivery, improved bioavailability, and long-term safety. Innovative strategies, including nano-carrier engineering and targeted probiotic preparations are expected to improve their bioavailability and tissue specificity. Future research should focus on clarifying the dose-response relationship, long-term safety, and establishing individualized treatment stratification based on patients’ microbiota-metabolic characteristics.

## 1 Introduction

Asthma is a chronic airway disease characterized by Th2-type inflammation, eosinophilic infiltration, and airway hyper reactivity(AHR). In recent years, the gut microbiota and its specific metabolic products, especially butyrate, have been shown to reshape the host immune system (1), and contribute to the pathogenesis of allergic airway diseases (2). Their regulatory roles in immunity have become a research hotspot (3). Epidemiological evidence suggests that early-life exposure to antibiotics and gut microbiota dysbiosis are significantly associated with increased asthma risk (4). Martin Depner and colleagues, through cohort studies and microbiome analyses, demonstrated that infants with farm exposure exhibited significantly higher gut microbial diversity during the first year of life compared to non-farm children. Moreover, greater microbial diversity was associated with a lower risk of asthma. These findings support a potential mechanism by which the “farm effect” reduces childhood asthma risk via a microbiota–SCFAs–immune regulatory axis (5). Gut microbiota may contribute to asthma protection through their metabolites, thereby supporting the concept of a “gut–lung axis” (6). Epidemiological data have also shown that reduced levels of three major SCFAs (butyrate, propionate, and acetate) in early life are associated with the development of atopic dermatitis (AD), wheezing/asthma, and IgE-mediated food allergies in childhood (7). As a key mediator of microbe–host interactions, butyrate exerts effects on asthma through various mechanisms, including epigenetic reprogramming and immune cell differentiation. This review summarizes the current research progress of the potential mechanisms and therapeutic implications of butyrate in asthma.

## 2 Inhibition of ILC2s overactivation

Allergic asthma is typically marked by AHR and chronic inflammation driven by aberrant Th2-type immune responses. ILC2s are pivotal effector cells in type 2 inflammation in asthma. Unlike adaptive Th2 cells, ILC2s can be directly activated by alarmins such as interleukin-33 (IL-33) and thymic stromal lymphopoietin (TSLP) (8), independent of antigen presentation. They are a key source of Th2 cytokines, particularly IL-5 and IL-13, which exacerbate acute asthma episodes (9). A team from Tianjin Medical University demonstrated that prenatal maternal exposure to antibiotics significantly reduces butyrate levels in neonatal gut microbiota. This depletion leads to an abnormal increase in pulmonary ILC2s frequency and activity, along with a downregulation of type I interferon (IFN-I) signaling, thereby increasing susceptibility to allergic airway inflammation later in life. The study further revealed that butyrate supplementation restores IFN-I signaling via activation of G-protein coupled receptor 41 (GPR41), thereby inhibiting ILC2s proliferation and the release of IL-13 and other cytokines, ultimately ameliorating the asthmatic phenotype in adulthood (4). Notably, maternal supplementation with butyrate during pregnancy reversed these effects and reduced asthma risk in offspring. These findings suggest that butyrate plays a critical role in modulating type 2 innate immunity during the neonatal period, highlighting a potential early-life intervention window for asthma prevention.Ahmed Kabil BASc and colleagues provided further experimental evidence that gut dysbiosis can reconfigure the immune cell interaction network—particularly the ILC2–B1 cell–innate IgE axis—leading to sustained allergic predisposition. Gut microbiota imbalance results in a decrease in SCFAs production, which triggers intestinal epithelial cells to release alarmins such as IL-25, IL-33, and TSLP. These factors activate ILC2s and enhance their secretion of IL-5 and IL-13, further promoting the proliferation and differentiation of B1 cells, a unique subset of innate-like B cells. Through a non-classical mechanism—independent of T cells or conventional antigen presentation—these B1 cells produce innate IgE. Unlike adaptive antigen-specific IgE, innate IgE is polyreactive and capable of recognizing a broad spectrum of allergens such as house dust mites and pollen. By binding to high-affinity IgE receptors (FcεRIα) on MCs or basophils, innate IgE induces degranulation and release of histamine and other inflammatory mediators, maintaining MCs in a pre-activated state. This sensitization can lead to low-threshold activation and recurrent allergic responses such as asthma or atopic dermatitis even in the absence of persistent antigen exposure. Furthermore, a decrease in Treg populations and dominance of Th2 responses facilitate the chronicity of this allergic predisposition (10).This body of work proposes a novel immunological pathway linking gut dysbiosis to systemic allergic susceptibility:

Microbiota dysbiosis → ILC2 activation → B1 cell differentiation → innate IgE production → MCs sensitization → allergic response. This mechanism extends beyond the classical Th2/adaptive immunity paradigm and provides new perspectives for both mechanistic studies and therapeutic interventions for allergic diseases. The latest study by Kaifan Bao’s team at Nanjing University of Chinese Medicine identified a novel memory-like subset of ILC2s (ml-ILC2s) with immunological memory features, phenotypically defined as CD45χlineage⁻CD90.2χNK1.1⁻NKp46⁻ST2⁻KLRG1χIL-17RBχ (11). This discovery reveals new mechanisms underlying recurrent asthma exacerbations. Ml-ILC2s possess migratory capacity between the lung and small intestine and regulate the episodic nature of asthma. These findings not only uncover the spatiotemporal dynamics of asthma relapses but also offer molecular evidence supporting the traditional Chinese medicine theory that “the lung and large intestine are externally–internally related.” ILC2s exhibit a “mirror-like” relationship with Th2 cells. Unlike Th2 cells, which require antigen-specific reactivation, ILC2s are rapidly triggered by nonspecific alarmins such as IL-25, IL-33, and TSLP (12). In both murine models and clinical samples, researchers found that during acute asthma exacerbations, ml-ILC2s proliferate extensively in the lung and later migrate to the lamina propria of small intestines (siLP) during remission, where they persist long-term under the regulation of the CCR9/CCL25 chemokine axis. These cells exhibit hallmarks of immune memory, including prolonged survival, heightened sensitivity, and amplified responses. Upon re-exposure to allergens or alarmins, ml-ILC2s are rapidly recruited back to the airway from the siLP via sphingosine-1-phosphate (S1P) and its receptors-S1PRs, where they release large quantities of IL-13, mediating more rapid and severe asthma relapses (11, 13). Further investigations revealed that the transcription factors T-cell-specific transcription factor-1 (TCF-1) and thymocyte selection-associated high mobility group box (TOX) are crucial for maintaining the immune memory and migratory function of ml-ILC2s (11).This research provides novel insights into the pathogenesis of asthma recurrence and identifies ml-ILC2s as a promising target for potential curative therapies.

Butyrate has been shown to ameliorate allergic airway inflammation by suppressing excessive activation of ILC2s. However, the mechanisms by which it regulates ILC2s metabolism remain incompletely understood. Current studies suggest the following potential mechanisms:

### 2.1 Epigenetic modifications

Butyrate modulates the epigenetic landscape of ILC2s by inhibiting histone deacetylases (HDACs) (9, 14). In particular, reduced HDAC3 activity results in increased chromatin acetylation. Through this mechanism, butyrate may suppress the transcription of pro-inflammatory genes such as those encoding IL-5 and IL-13 in ILC2s (15), while simultaneously enhancing the expression of anti-inflammatory genes, including Forkhead Box Protein P3 (Foxp3), thereby indirectly mitigating ILC2-driven inflammatory responses (16) and attenuating ILC2-associated long-term immune memory activity. In the study by Jiang et al., butyrate selectively inhibits HDAC activity in ILC2s, inducing histone hyperacetylation. This epigenetic modification enhances chromatin accessibility at specific gene promoter regions, leading to robust upregulation of the transcription factor nuclear factor interleukin-3 regulated (NFIL3). NFIL3 directly binds to effector gene loci (e.g., Il5, Il13) in ILC2s, repressing their transcription. Furthermore, NFIL3 suppresses expression of GATA binding protein 3(GATA3)—the master regulator of ILC2s differentiation and maintenance (17, 18). Notably, a study by Theresa Alenghat’s group, published in Immunity in January 2024, demonstrated that HDAC3 deficiency significantly diminished type 2 immune responses during helminth infection in murine models. Furthermore, treatment with either butyrate or HDAC3 inhibitors suppressed the expansion of tuft cells in human intestinal organoids and mouse models, reducing IL-25 secretion—a critical activator of ILC2s—thereby further inhibiting ILC2s activation and the downstream type 2 immune response. These findings underscore the central role of HDAC3 in the epigenetic regulation of ILC2s (19).

### 2.2 Regulation of receptor-mediated signaling pathways

Butyrate modulates immune cell function by engaging G protein-coupled receptors (GPCRs), e.g., GPR41 and GPR43 (20), which are expressed by ILC2s, rendering them directly responsive to butyrate. In maternal antibiotic exposure models, butyrate deficiency leads to aberrant ILC2s activation, whereas supplementation restores ILC2s homeostasis via a GPR41-dependent mechanism. In GPR41-deficient mice, the anti-inflammatory effects of butyrate are abolished, with significantly elevated IL-5 and IL-13 production by ILC2s (4). Upon GPCR activation, butyrate may inhibit nuclear factor kappa-B (NF-κB) signaling via the cAMP–PKA pathway, thereby reducing the secretion of pro-inflammatory cytokines from ILC2s (21), and exerting immunosuppressive effects.

### 2.3 Enhancement of IFN-I signaling

The activation of ILC2s is negatively regulated by IFN-I signaling (22). Butyrate enhances the expression of interferon alpha and beta receptor 1 (IFNAR1), leading to increased phosphorylation of the downstream signaling molecule signal transducers and activators of transcription 1 (STAT1), thereby counteracting ILC2-mediated type 2 inflammation (4, 23). Animal experiments showed that IFNAR1 knockout mice were unable to reverse ILC2s hyperactivation after butyrate intervention, further demonstrating the critical role of this pathway in immune regulation (4). Current evidence supports that IFN-I suppresses Th2 responses and enhances immune function via the IFNAR1–STAT1 axis (4, 24), and that butyrate can further strengthen this pathway through metabolic-immune cross-talk.

### 2.4 Inhibition of PPARγ signaling affecting fatty acid oxidation and lipid metabolism

Although no study has directly examined the effects of peroxisome proliferator-activated receptor gamma (PPARγ) antagonists or gene knockout on ILC2s function, findings from models involving PPARγ in butyrate metabolism, immune regulation, and tissue-specific knockout in the liver and neural stem cells (25) support the following hypothesis, which may serve as an indirect rationale and potential future research direction: The activation of ILC2s and the production of their key effector cytokines (e.g., IL-5, IL-13) are highly dependent on lipid metabolism, particularly FAO and lipid droplet formation. Butyrate may suppress PPARγ signaling by downregulating its expression, thereby limiting lipid storage and the availability of substrates for fatty acid oxidation. This, in turn, may impair lipid droplet biogenesis and effector functions in ILC2s (16, 25, 26). Future studies should consider developing ILC2-specific conditional PPARγ knockout mouse models—following strategies used in neural stem cell or hepatic-specific knockout models—or employing PPARγ antagonists (e.g., GW9662) in combination with butyrate treatment to test whether butyrate downregulates PPARγ to modulate ILC2s metabolic status and immune activity.

## 3 Inhibition of MCs activation mediated via the PGE2–EP3 signaling axis

Recent studies have demonstrated that butyrate exerts inhibitory effects on MCs activation through engagement of the G protein-coupled receptor 109A (GPR109A), subsequently initiating downstream signaling via the Gi protein cascade. This signaling promotes the release of prostaglandin E2 (PGE2), which then binds to the recombinant prostaglandin E receptor 3 (EP3), thereby inhibiting MCs degranulation and the release of pro-inflammatory mediators. Further experimental evidence reveals that the anti-allergic effects of SCFAs can be abrogated by cyclooxygenase (COX) inhibitors (e.g., aspirin) or EP3 antagonists, underscoring the central role of PGE2 in this pathway. This mechanism has also been validated in animal models; in a murine passive cutaneous anaphylaxis model, oral administration of butyrate or niacin (a known high-affinity GPR109A ligand) significantly inhibited IgE-mediated MCs degranulation, reduced vascular leakage in the skin, and ameliorated anaphylactic symptoms. These effects are dependent on the GPR109A–PGE2 signaling axis and are regulated by the COX pathway (27).

## 4 Epigenetic regulatory of butyrate

Butyrate, as classical HDAC inhibitors, markedly increase histone acetylation levels, thereby relaxing chromatin architecture and enhancing the transcriptional activity of specific genes, ultimately influencing immune cell differentiation (28). By inhibiting HDAC activity and increasing histone acetylation, butyrate promotes the expression of anti-inflammatory genes (e.g., Foxp3), while suppressing the transcription of pro-inflammatory genes (e.g., the IL-33 receptor ST2), thus effectively attenuating airway inflammation. In asthmatic mice, Ramiya Islam et al. (29) demonstrated that intranasal administration of sodium butyrate or a broad-spectrum HDAC inhibitor significantly alleviated asthma symptoms by suppressing HDAC1 expression. The study further proposed that butyrate may inhibit HDAC1 via downregulation of the p-Akt/p-PI3K/HIF-1α/VEGF signaling axis, thereby dampening airway inflammation. Ravindra Gudneppanavar et al. (30) further revealed that butyrate, as an HDAC inhibitor, exerts its regulatory effect not through the canonical G protein-coupled receptors GPR41 (FFAR3) or GPR43 (FFAR2), but rather via histone modification-dependent suppression of the v-kit Hardy-Zuckerman 4 feline sarcoma viral oncogene homolog (c-Kit) gene transcription. This results in reduced phosphorylation of downstream p38 and extracellular signal-regulated kinase (Erk) pathways, thereby inhibiting MCs activation and function. Elevated histone acetylation levels generally promote chromatin decondensation and facilitate transcription factor binding, activating gene expression. However, under certain conditions, acetylation may also mediate transcriptional repression via multiple mechanisms. For instance, c-Kit encodes the stem cell factor (SCF) receptor, a pivotal regulator of MCs survival, proliferation, and activation. Activation of the c-Kit axis drives MCs degranulation and the release of histamine, cytokines (e.g., IL-6, TNF-α), and proteases, contributing to allergic and inflammatory responses (31). Butyrate-induced hyperacetylation of histones H3 and H4 in the c-Kit promoter region can suppress its transcription through the following mechanisms: First, by recruiting transcriptional repressor complexes and modulating promoter activity: hyperacetylation may expose repressive cis-elements within the KIT gene (also known as c-kit) promoter, facilitating the recruitment of silencing complexes such as the H3K27me3-dependent Polycomb Repressive Complex 2 (PRC2), thereby silencing c-Kit expression (30, 32). Second, by inducing expression of repressive transcription factors: butyrate can upregulate transcriptional repressors such as Foxp3 and PPARγ, which bind to the c-Kit promoter and inhibit its transcriptional activity (30). Third, by activating repressive non-coding RNAs: histone acetylation may promote the expression of long non-coding RNAs (lncRNAs) or microRNAs (miRNAs) that target c-Kit mRNA or its promoter, thereby inhibiting its expression or stability (30–32). Suppression of the c-Kit signaling pathway can induce MCs apoptosis or cell cycle arrest, resulting in reduced degranulation and decreased release of inflammatory mediators such as histamine and proteases, ultimately alleviating allergic responses (33). The decrease in pro-inflammatory cytokines (e.g., IL-6, TNF-α) may be accompanied by an increase in anti-inflammatory mediators (e.g., IL-10), contributing to the reestablishment of immune homeostasis (31, 32). Furthermore, Cecilia Lövkvist et al. (30) proposed the concept of epigenetic “memory,” whereby histone modifications induced by butyrate may persist even after its removal. This memory relies on positive feedback loops between repressive complexes (e.g., PRC2) and histone marks (e.g., H3K27me3), enabling sustained silencing of target genes and providing a mechanistic basis for the long-term immunomodulatory effects of butyrate.

Taken together, butyrate inhibit HDAC activity, enhance histone acetylation, and recruit repressive complexes and transcriptional regulators to cooperatively downregulate c-Kit expression and suppress MCs function. This process integrates epigenetic regulation, non-coding RNA interference, and immunometabolic reprogramming, establishing a multi-mechanistic, cross-organ model of immune modulation. Butyrate represents a promising epigenetic therapeutic strategy for targeting c-Kit expression, potentially circumventing resistance issues associated with traditional antihistamines. Additionally, butyrate produced by the gut microbiota may regulate MCs function in distant tissues via the “gut-immune axis” (27, 34), offering novel insights for dietary-based preventive and therapeutic approaches.

Butyrate modulate epigenetic modifications by inhibiting HDACs and downregulate the expression of the high-affinity IgE receptor (FcϵRI) on the surface of MCs, thereby reducing their sensitivity to IgE-mediated stimuli. This mechanism effectively suppresses MCs degranulation and the release of inflammatory mediators such as histamine and leukotrienes, ultimately alleviating allergic and inflammatory responses. Studies have shown that the HDAC inhibitor Trichostatin A (TSA) mimics the effects of SCFAs in this pathway, significantly attenuating FcϵRI-mediated intracellular signaling, highlighting the potential application of such epigenetic modulators in the treatment of allergic diseases (27).

The epigenetic regulation exerted by butyrate intersects with other epigenetic mechanisms. As a microbial metabolite, butyrate not only modulates gene expression through its known role as an HDAC inhibitor, but also affects intracellular acetyl-CoA levels via metabolic reprogramming, thereby regulating histone acetylation status. Studies have demonstrated that butyrate can be oxidized to generate acetyl-CoA, which serves as a substrate for histone acetyltransferases (HATs), thereby promoting histone acetylation, chromatin accessibility, and gene expression (35). Moreover, butyrate acts synergistically with other epigenetic modifications to form a complex regulatory network. A recent study published in Cell identified histone lactylation as a novel epigenetic mark capable of maintaining transcriptional memory during metabolic stress and contributing to trained immunity. Inhibition of lactate production or histone lactylation was shown to abolish trained immune responses, underscoring the critical role of this modification in maintaining immune memory (36), which may synergize with c-Kit suppression. Additionally, butyrate may inhibit DNA methyltransferases (DNMTs), reducing promoter methylation of specific genes and further modulating gene expression (37, 38). For example, butyrate can induce demethylation of the retinoic acid receptor β2 (RARβ2) gene promoter, enhancing its expression. Notably, this demethylation differs from that mediated by classical DNMT inhibitors, being promoter-specific, genome-sparing, and replication-independent (39).

Butyrate affects the epigenetic state through a variety of mechanisms, including regulating acetyl-CoA level, promoting histone acetylation, participating in histone lactamation, and inhibiting DNA methylation, forming a complex metabolism-epigenetic regulatory network. These mechanisms play an important role in maintaining intestinal homeostasis, regulating immune responses, and possibly antitumor effects. However, the priority of butyrate among different epigenetic modifications and the specific synergistic mechanism still need to be further studied to clarify its potential application value in the treatment of diseases.

**Figure 1:**
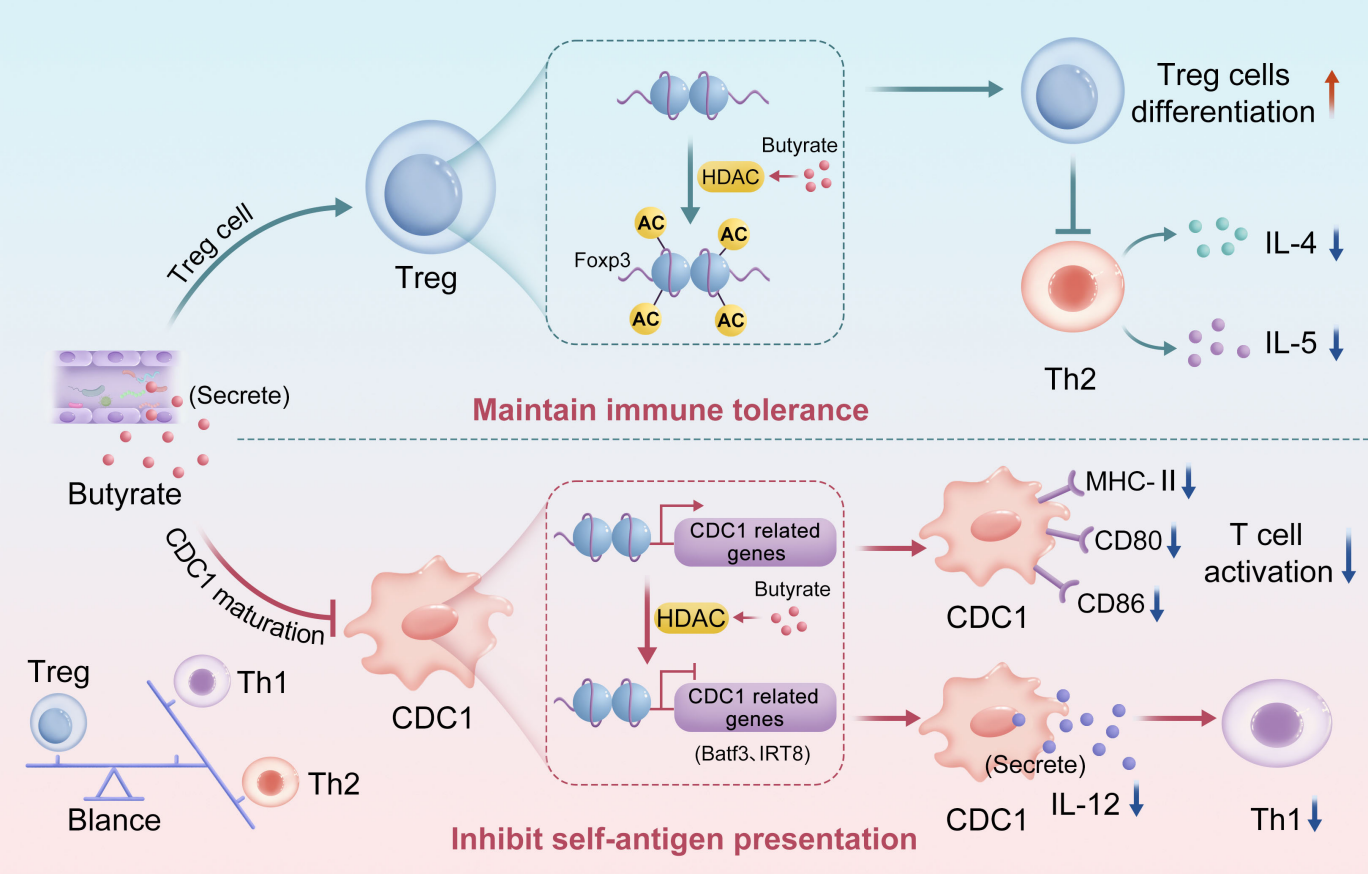
Schematic representation of dual regulation of immunity by butyrate through epigenetics.

Butyrate engage in bidirectional regulation of the immune system via epigenetic pathways to maintain dynamic homeostasis (19), particularly in Th2-dominant allergic diseases such as asthma. On the one hand, butyrate inhibits HDACs and increases histone H3 acetylation at the Foxp3 promoter, thereby promoting the differentiation and function of Treg cells. Studies have demonstrated that butyrate significantly upregulates Foxp3 expression, enhances the immunosuppressive capacity of Treg cells, and suppresses Th2-mediated inflammation by reducing cytokines such as IL-4 and IL-5, thereby maintaining immune tolerance (4, 40). On the other hand, butyrate impairs the development and function of classical dendritic cells (cDCs), which are professional antigen-presenting cells essential for T cell activation and adaptive immunity (41, 42). Butyrate may downregulate the expression of transcription factors critical for cDC differentiation, such as interferon regulatory factor 8 (IRF8) and basic leucine zipper transcriptional factor ATF-like 3 (Batf3), through HDAC inhibition, thereby impeding cDC maturation (26, 42, 43). Furthermore, butyrate downregulates the expression of major histocompatibility complex class II (MHC-II) and costimulatory molecules (e.g., CD80, CD86) on cDCs, diminishing their antigen-presenting capacity and T cell priming efficiency (19, 42, 44). Given that cytokines such as IL-12 produced by cDCs are crucial for T helper 1 (Th1) cell differentiation, butyrate may reduce their production via HDAC inhibition, impairing Th1 activation and further influencing the Th1/Th2/Treg balance (45) (**Figure 1**).

By promoting the differentiation of Treg cells (acting as an “immune brake”) and inhibiting cDC function (weakening the “immune throttle”), butyrate forms a dynamic balance in the immune system and prevents excessive activation or suppression of the immune response. This mechanism is particularly critical in Th2-dominated diseases such as asthma, suggesting that regulation of gut microbiota metabolites, especially butyrate, may provide a new strategy for the treatment of immune-related diseases.

## 5 The role of TSLP in airway inflammation and the potential mechanisms of butyrate intervention

TSLP is a critical epithelial-derived cytokine that orchestrates airway inflammation by activating downstream immune cells, including eosinophils, neutrophils, and ILC2s. TSLP induces the expression of glucocorticoid receptor-β (GRβ), a structural homolog of the classical glucocorticoid receptor-α (GRα), which lacks ligand-binding capacity and functions as a dominant-negative inhibitor of GRα-mediated transcription. The upregulation of GRβ attenuates glucocorticoid responsiveness, leading to corticosteroid resistance (CR) (46). Moreover, elevated GRβ expression is associated with prolonged survival of eosinophils and neutrophils, which continuously secrete pro-inflammatory cytokines such as IL-5 and IL-13, exacerbating inflammation and resistance. These findings underscore TSLP as a pivotal mediator of CR via GRβ induction in airway inflammatory diseases. Emerging evidence has identified elevated TSLP levels in asthma and chronic obstructive pulmonary disease (COPD), where it exacerbates inflammation and steroid insensitivity through the activation of Th2 cell responses and eosinophilic infiltration (47, 48).

Recent studies from the Beijing Institute of Animal Science and Veterinary Medicine suggest that butyrate, through its metabolite naringenin, can attenuate inflammatory responses by suppressing the PI3K-Akt and NF-κB signaling pathways and enhancing the expression of tight junction proteins in the intestinal epithelium (49). Although this research focused on necrotizing enterocolitis (NEC), the PI3K-Akt and NF-κB pathways are likewise crucial regulators of TSLP expression. For instance, TSLP has been shown to activate downstream inflammatory cascades via NF-κB, with elevated TSLP levels in ovalbumin (OVA)-induced asthma models closely associated with NF-κB nuclear translocation (50). TSLP is also a central mediator of type 2 immunity, including anti-helminth responses and allergic airway inflammation. It promotes Th2 cell differentiation by activating dendritic cell (DC) surface receptors such as CD40, CD80, and CD86, leading to increased secretion of IL-4, IL-5, and IL-13, thereby aggravating airway inflammation (51). Anti-TSLP therapies such as Tezspire suppress TSLP signaling, reduce peripheral eosinophil counts, and significantly diminish Th2 cytokine release, improving airway inflammation and reducing the frequency of acute exacerbations in severe asthma (52, 53), thereby validating TSLP’s central role in Th2-driven pathology. Additionally, butyrate has been shown to inhibit Th2 cell function and diminish the release of pro-inflammatory cytokines. It also promotes the differentiation of Tregs, which can indirectly suppress Th2-mediated inflammation. This suggests that Th2 cells may represent a shared target through which both TSLP and butyrate exert their immunomodulatory effects. To date, no direct evidence has been reported on the ability of butyrate to suppress TSLP expression in airway epithelial cells. However, the anti-inflammatory mechanisms of butyrate—namely inhibition of the NF-κB and PI3K-Akt pathways and suppression of Th2 cytokine production—substantially overlap with the signaling axes that regulate TSLP expression. Future studies should investigate whether butyrate downregulates TSLP transcription in airway epithelial cells via these shared pathways, thereby interrupting the TSLP-DC-Th2 axis. If validated, butyrate may represent a novel anti-inflammatory intervention strategy and offer new avenues for the development of more effective and stable asthma control therapies.

## 6 Modulation of the gut–lung axis

The gut microbiota exerts distal immunoregulatory effects on pulmonary immune homeostasis through the production of SCFAs, particularly butyrate. A pioneering study by Nanjing University of Chinese Medicine first revealed that ILC2s can migrate from the lung to the intestinal lamina propria via the CCR9/CCL25 chemokine axis—termed lung-to-gut migration—where they differentiate into memory-like ILC2s subsets (ml-ILC2s) (11). Butyrate maintains gut microbial balance and downregulates CCL25 expression, thereby disrupting the CCR9/CCL25 signaling axis. This limits the intestinal residence and pulmonary recruitment of ml-ILC2s, ultimately interrupting the recurrence cycle of asthma (11, 54). These findings expand the current understanding of mucosal immune regulation across organs and suggest that the gut may serve as a distal immunological hub for respiratory diseases. Furthermore, the study holds significant translational potential: serum CCL25 levels may serve as a predictive biomarker for asthma recurrence.

Maintenance of Gut Barrier Integrity (55, 56) and Distal Modulation of Pulmonary Inflammation: SCFAs promote the expression of tight junction proteins in intestinal epithelial cells, such as claudin-1 and occludin, thereby enhancing gut barrier function and reducing intestinal permeability. This limits the translocation of bacterial metabolites (e.g., lipopolysaccharide, LPS) into the systemic circulation, subsequently mitigating systemic inflammation and indirectly inhibiting the recruitment of pulmonary ILC2s (57). A research team from Sichuan University developed a butyrate-derived polymeric nanoparticle platform (PSBA@Mag) that demonstrated significant efficacy in a colitis model. This nanoplatform enables pH/redox dual-responsive release of butyrate and the anti-inflammatory phytochemical magnolol, thereby restoring the expression of claudin-1 and occludin, repairing the intestinal epithelial barrier, and rebalancing the gut microbiota to alleviate inflammation (58). In addition, Xu Gong and colleagues at Beihang University demonstrated that butyrate acts as an HDAC3 inhibitor to suppress expression of Gasdermin D (GSDMD), a key executor of pyroptosis in colonic epithelial cells. This protects the intestinal mucosa from inflammatory damage and prevents proinflammatory mediators from entering the bloodstream, thereby indirectly regulating pulmonary inflammation at a distal site (59).

The above studies provide a new perspective for the secondary prevention of asthma, and extend the traditional treatment strategy limited to the respiratory tract to the regulation of intestinal microecology, which is in line with the treatment concept of “holistic medicine”. The regulation of intestinal microenvironment in the remission stage of asthma may be more effective than the intervention in the acute stage. In the future, it is necessary to verify the results of animal studies in human trials, and to explore the optimal dose and route of administration of butyrate, such as oral formulations or rectal administration.

## 7 Summary and perspectives

Maternal-infant cohort studies have demonstrated that perinatal supplementation with butyrate significantly reduces the incidence of asthma in offspring. In animal models, prenatal administration of butyrate reverses antibiotic-induced epigenetic abnormalities in ILC2s and improves pulmonary function in adulthood. These findings suggest that the prenatal and neonatal periods may represent novel therapeutic windows for asthma intervention (4). Early-life butyrate supplementation shows considerable therapeutic potential and translational value. Butyrate exerts anti-asthmatic effects through multiple mechanisms, including modulation of immune cell function, epigenetic regulation, receptor signaling pathways, and gut–lung axis interactions. Its multi-target and bidirectional regulatory characteristics offer new therapeutic directions for allergic diseases. Future research should integrate metabolomics and single-cell sequencing to further delineate the direct molecular targets and regulatory networks of butyrate, thereby advancing precision therapeutic strategies based on microbial metabolites. For instance, the development of butyrate derivatives that specifically activate GPR109A or inhibit HDACs may reduce systemic side effects (27). We propose several key challenges and directions for future investigations: First, dosage and delivery: Precise regulation of local intestinal concentration and systemic exposure of butyrate is required to avoid adverse effects such as intestinal acidification. Second, individual variability: Host genetic background, gut microbiota composition, and asthma endotypes may influence therapeutic efficacy, necessitating the identification of biomarkers for stratified treatment. Third, validation of combination therapies: The synergistic effects of butyrate with probiotics, anti-IgE antibodies, or Treg inducers—as well as its co-administration with current biologics such as anti-IL-4R monoclonal antibodies—should be investigated to determine whether such strategies enhance the modulation of airway remodeling in asthma patients.

Lastly, exploration of traditional Chinese medicine (TCM) efficacy: Existing studies suggest that TCM formulations such as *Shenling Baizhu Powder* and *Lizhong Decoction* restore the diversity and community structure of butyrate-producing bacteria in antibiotic-associated diarrhea (AAD) rat models through their polysaccharide components, thereby enhancing butyrate production and improving gut microbiota function (60, 61). This selective modulatory effect may be related to the complex structural features of TCM polysaccharides (e.g., glycosidic linkage types and degrees of branching). The underlying molecular mechanisms are likely to involve interactions between microbial metabolites and host epigenetic modifications. Several studies further indicate that SCFAs may serve as critical targets in the treatment of diseases by TCM formulations. However, the precise mechanisms remain to be elucidated, and further experimental and clinical studies are required to validate whether TCM formulations can serve as a safe and effective strategy for butyrate supplementation. This review highlights butyrate—a natural products—as a promising anti-asthmatic agent capable of modulating the immune microenvironment, regulating gut–lung interactions, and reducing corticosteroid dependence through multifaceted mechanisms. Future strategies may include dietary interventions, microbiome engineering, or TCM-based approaches to optimize asthma management. Such endeavors may pave the way for metabolite-based precision therapies and open new avenues for the prevention of asthma.

## References

[1] Kim CH. Complex regulatory effects of gut microbial short-chain fatty acids on immune tolerance and autoimmunity. Cell Mol Immunol. (2023) 20:341–50. doi: 10.1038/s41423-023-00987-1, PMID: 36854801 PMC10066346

[2] Mann ER Lam YK Uhlig HH. Short-chain fatty acids: linking diet, the microbiome and immunity. Nat Rev Immunol. (2024) 24:577–95. doi: 10.1038/s41577-024-01014-8, PMID: 38565643

[3] Yip W Hughes MR Li Y Cait A Hirst M Mohn WW. Butyrate shapes immune cell fate and function in allergic asthma. Front Immunol. (2021) 12:628453. doi: 10.3389/fimmu.2021.628453, PMID: 33659009 PMC7917140

[4] Xu H Yi X Cui Z Li H Zhu L Zhang L. Maternal antibiotic exposure enhances ILC2 activation in neonates via downregulation of IFN1 signaling. Nat Commun. (2023) 14:8332. doi: 10.1038/s41467-023-43903-x, PMID: 38097561 PMC10721923

[5] Depner M Taft DH Kirjavainen PV Kalanetra KM Karvonen AM Peschel S. Maturation of the gut microbiome during the first year of life contributes to the protective farm effect on childhood asthma. Nat Med. (2020) 26:1766–75. doi: 10.1038/s41591-020-1095-x, PMID: 33139948

[6] Singh V Lee G Son H Koh H Kim ES Unno T. Butyrate producers, “The Sentinel of Gut”: Their intestinal significance with and beyond butyrate, and prospective use as microbial therapeutics. Front Microbiol. (2023) 13:1103836. doi: 10.3389/fmicb.2022.1103836, PMID: 36713166 PMC9877435

[7] Sasaki M Suaini NHA Afghani J Heye KN O'Mahony L Venter C. Systematic review of the association between short-chain fatty acids and allergic diseases. Allergy. (2024) 79:1789–811. doi: 10.1111/all.16065, PMID: 38391245

[8] Akenroye A Boyce JA Kita H. Targeting alarmins in asthma: From bench to clinic. J Allergy Clin Immunol. (2025) 155:1133–48. doi: 10.1016/j.jaci.2025.01.017, PMID: 39855362 PMC12555011

[9] Thio CL Chi PY Lai AC Chang YJ. Regulation of type 2 innate lymphoid cell-dependent airway hyperreactivity by butyrate. J Allergy Clin Immunol. (2018) 142:1867–1883.e12. doi: 10.1016/j.jaci.2018.02.032, PMID: 29522844

[10] Kabil A Nayyar N Brassard J Li Y Chopra S Hughes MR. Microbial intestinal dysbiosis drives long-term allergic susceptibility by sculpting an ILC2-B1 cell-innate IgE axis. J Allergy Clin Immunol. (2024) 154:1260–1276.e9. doi: 10.1016/j.jaci.2024.07.023, PMID: 39134158

[11] Bao K Gu X Song Y Zhou Y Chen Y Yu X. TCF-1 and TOX regulate the memory formation of intestinal group 2 innate lymphoid cells in asthma. Nat Commun. (2024) 15:7850. doi: 10.1038/s41467-024-52252-2, PMID: 39245681 PMC11381517

[12] Gurram RK Wei D Yu Q Butcher MJ Chen X Cui K. Crosstalk between ILC2s and Th2 cells varies among mouse models. Cell Rep. (2023) 42:112073. doi: 10.1016/j.celrep.2023.112073, PMID: 36735533 PMC10394112

[13] Lan F Li J Miao W Sun F Duan S Song Y. GZMK-expressing CD8+ T cells promote recurrent airway inflammatory diseases. Nature. (2025) 638:490–8. doi: 10.1038/s41586-024-08395-9, PMID: 39814882 PMC11821540

[14] Kabata H Moro K Koyasu S. The group 2 innate lymphoid cell (ILC2) regulatory network and its underlying mechanisms. Immunol Rev. (2018) 286:37–52. doi: 10.1111/imr.12706, PMID: 30294963

[15] Cardoso V Chesné J Ribeiro H García-Cassani B Carvalho T Bouchery T. Neuronal regulation of type 2 innate lymphoid cells via neuromedin U. Nature. (2017) 549:277–81. doi: 10.1038/nature23469, PMID: 28869974 PMC5714273

[16] Zhou L Lin Q Sonnenberg GF. Metabolic control of innate lymphoid cells in health and disease. Nat Metab. (2022) 4:1650–9. doi: 10.1038/s42255-022-00685-8, PMID: 36424470 PMC9789197

[17] Jiang M Wang J Li Z Xu D Jing J Li F. Dietary fiber-derived microbial butyrate suppresses ILC2-dependent airway inflammation in COPD. Mediators Inflamm. (2024) 2024:6263447. doi: 10.1155/2024/6263447, PMID: 39015676 PMC11251798

[18] Zhang X Liu J Li X Zheng G Wang T Sun H. Blocking the HIF-1α/glycolysis axis inhibits allergic airway inflammation by reducing ILC2 metabolism and function. Allergy. (2025) 80:1309–34. doi: 10.1111/all.16361, PMID: 39462230

[19] Eshleman EM Rice T Potter C Waddell A Hashimoto-Hill S Woo V. Microbiota-derived butyrate restricts tuft cell differentiation via histone deacetylase 3 to modulate intestinal type 2 immunity. Immunity. (2024) 57:319–332.e6. doi: 10.1016/j.immuni.2024.01.002, PMID: 38295798 PMC10901458

[20] Lee DH Kim MT Han JH. GPR41 and GPR43: From development to metabolic regulation. BioMed Pharmacother. (2024) 175:116735. doi: 10.1016/j.biopha 38744220

[21] Xiao Q Han X Liu G Zhou D Zhang L He J. Adenosine restrains ILC2-driven allergic airway inflammation via A2A receptor. Mucosal Immunol. (2022) 15:338–50. doi: 10.1038/s41385-021-00475-7, PMID: 34921233

[22] Reverter A Ballester M Alexandre PA Mármol-Sánchez E Dalmau A Quintanilla R. A gene co-association network regulating gut microbial communities in a Duroc pig population. Microbiome. (2021) 9:52. doi: 10.1186/s40168-020-00994-8, PMID: 33612109 PMC7898758

[23] Jung MA Lee JY Kim YJ Ji KY Le MH Jung DH. Inula japonica Thunb. and its active compounds ameliorate airway inflammation by suppressing JAK-STAT signaling. BioMed Pharmacother. (2025) 183:117852. doi: 10.1016/j.biopha.2025.117852, PMID: 39854818

[24] Ma H Yang W Zhang L Liu S Zhao M Zhou G. Interferon-alpha promotes immunosuppression through IFNAR1/STAT1 signalling in head and neck squamous cell carcinoma. Br J Cancer. (2019) 120:317–30. doi: 10.1038/s41416-018-0352-y, PMID: 30555157 PMC6353953

[25] Wang H Zhang X Xue QL Han SC Li WH Zhao TT. Protective effect and mechanism of seabuckthorn polysaccharides on sepsis-induced liver injury in liver-specific PPARγ knockout mice. Chin J Immunol. (2022) 38:789–94.

[26] Wang R Li B Huang B Li Y Liu Q Lyu Z. Gut microbiota-derived butyrate induces epigenetic and metabolic reprogramming in myeloid-derived suppressor cells to alleviate primary biliary cholangitis. Gastroenterology. (2024) 167:733–749.e3. doi: 10.1053/j.gastro.2024.05.014, PMID: 38810839

[27] Nagata K Ando D Ashikari T Ito K Miura R Fujigaki I. Butyrate, valerate, and niacin ameliorate anaphylaxis by suppressing igE-dependent mast cell activation: roles of GPR109A, PGE2, and epigenetic regulation. J Immunol. (2024) 212:771–84. doi: 10.4049/jimmunol.2300188, PMID: 38197634

[28] Artusa V Calabrone L Mortara L Peri F Bruno A. Microbiota-derived natural products targeting cancer stem cells: inside the gut pharma factory. Int J Mol Sci. (2023) 24:4997. doi: 10.3390/ijms24054997, PMID: 36902427 PMC10003410

[29] Islam R Dash D Singh R. An antioxidant ameliorates allergic airway inflammation by inhibiting HDAC 1 via HIF-1α/VEGF axis suppression in mice. Sci Rep. (2023) 13:9637. doi: 10.1038/s41598-023-36678-0, PMID: 37316684 PMC10267105

[30] Gudneppanavar R Sabu Kattuman EE Teegala LR Southard E Tummala R Joe B. Epigenetic histone modification by butyrate downregulates KIT and attenuates mast cell function. J Cell Mol Med. (2023) 27:2983–94. doi: 10.1111/jcmm.17924, PMID: 37603611 PMC10538265

[31] Wu J Zhao Y Wang X Kong L Johnston LJ Lu L. Dietary nutrients shape gut microbes and intestinal mucosa via epigenetic modifications. Crit Rev Food Sci Nutr. (2022) 62:783–97. doi: 10.1080/10408398.2020.1828813, PMID: 33043708

[32] Ye J Wu W Li Y Li L. Influences of the gut microbiota on DNA methylation and histone modification. Dig Dis Sci. (2017) 62:1155–64. doi: 10.1007/s10620-017-4538-6, PMID: 28341870

[33] Wu D Shi Y Zhang H Miao C. Epigenetic mechanisms of Immune remodeling in sepsis: targeting histone modification. Cell Death Dis. (2023) 14:112. doi: 10.1038/s41419-023-05656-9, PMID: 36774341 PMC9922301

[34] Lövkvist C Mikulski P Reeck S Hartley M Dean C Howard M. Hybrid protein assembly-histone modification mechanism for PRC2-based epigenetic switching and memory. Elife. (2021) 10:e66454. doi: 10.7554/eLife.66454.sa2, PMID: 34473050 PMC8412945

[35] Egger AS Rauch E Sharma S Kipura T Hotze M Mair T. Linking metabolism and histone acetylation dynamics by integrated metabolic flux analysis of Acetyl-CoA and histone acetylation sites. Mol Metab. (2024) 90:102032. doi: 10.1016/j.molmet.2024.102032, PMID: 39305948 PMC11492620

[36] Ziogas A Novakovic B Ventriglia L Galang N Tran KA Li W. Long-term histone lactylation connects metabolic and epigenetic rewiring in innate immune memory. Cell. (2025) 188(11):2992-3012.e16. doi: 10.1016/j.cell.2025.03.048, PMID: 40318634

[37] Li H Ryu MH Rider CF Tse W Clifford RL Aristizabal MJ. Predominant DNMT and TET mediate effects of allergen on the human bronchial epithelium in a controlled air pollution exposure study. J Allergy Clin Immunol. (2021) 147:1671–82. doi: 10.1016/j.jaci.2020.08.044, PMID: 33069714

[38] Chang X Ma J Xue X Wang G Yan T Su L. DNMT family induces down-regulation of NDRG1 via DNA methylation and clinicopathological significance in gastric cancer. PeerJ. (2021) 9:e12146. doi: 10.7717/peerj.12146, PMID: 34616614 PMC8450010

[39] Spurling CC Suhl JA Boucher N Nelson CE Rosenberg DW Giardina C. The short chain fatty acid butyrate induces promoter demethylation and reactivation of RARbeta2 in colon cancer cells. Nutr Cancer. (2008) 60:692–702. doi: 10.1080/01635580802008278, PMID: 18791934

[40] Martin-Gallausiaux C Béguet-Crespel F Marinelli L Jamet A Ledue F Blottière HM. Butyrate produced by gut commensal bacteria activates TGF-beta1 expression through the transcription factor SP1 in human intestinal epithelial cells. Sci Rep. (2018) 8:9742. doi: 10.1038/s41598-018-28048-y, PMID: 29950699 PMC6021401

[41] Jirmo AC Grychtol R Gaedcke S Liu B DeStefano S Happle C. Single cell RNA sequencing reveals distinct clusters of Irf8-expressing pulmonary conventional dendritic cells. Front Immunol. (2023) 14:1127485. doi: 10.3389/fimmu.2023.1127485, PMID: 37251386 PMC10213693

[42] Andrusaite A Lewis J Frede A Farthing A Kästele V Montgomery J. Microbiota-derived butyrate inhibits cDC development via HDAC inhibition, diminishing their ability to prime T cells. Mucosal Immunol. (2024) 17:1199–211. doi: 10.1016/j.mucimm.2024.08.003, PMID: 39142634 PMC11631772

[43] Zhang Y Wu T He Z Lai W Shen X Lv J. Regulation of pDC fate determination by histone deacetylase 3. Elife. (2023) 12:e80477. doi: 10.7554/eLife.80477, PMID: 38011375 PMC10732571

[44] Eshleman EM Alenghat T. Epithelial sensing of microbiota-derived signals. Genes Immun. (2021) 22:237–46. doi: 10.1038/s41435-021-00124-w, PMID: 33824498 PMC8492766

[45] Niu J Cui M Yang X Li J Yao Y Guo Q. Microbiota-derived acetate enhances host antiviral response via NLRP3. Nat Commun. (2023) 14:642. doi: 10.1038/s41467-023-36323-4, PMID: 36746963 PMC9901394

[46] Xue JM An YF Suo LM Mo LH Yang G Luo XQ. Livin in synergy with Ras induces and sustains corticosteroid resistance in the airway mucosa. Int J Biol Sci. (2021) 17:2089–98. doi: 10.7150/ijbs.58427, PMID: 34131408 PMC8193260

[47] Vaghi A Bilò MB Bini F Cecchi L Micheletto C Musarra A. The added value of targeting airway hyperresponsiveness by blocking TSLP in the management of severe asthma. Eur Ann Allergy Clin Immunol. (2024). doi: 10.23822/EurAnnACI.1764-1489.376, PMID: 39545827

[48] Li Y Zhou Y Liu L Yang Y Liu Y Yan D. Osthole attenuates asthma-induced airway epithelial cell apoptosis and inflammation by suppressing TSLP/NF-κB-mediated inhibition of Th2 differentiation. Allergy Asthma Clin Immunol. (2024) 20:51. doi: 10.1186/s13223-024-00913-8, PMID: 39334402 PMC11438018

[49] Gao Y Yang L Yao Q Wang J Zheng N. Butyrate improves recovery from experimental necrotizing enterocolitis by metabolite hesperetin through potential inhibition the PI3K-Akt pathway. BioMed Pharmacother. (2024) 176:116876. doi: 10.1016/j.biopha.2024.116876, PMID: 38850657

[50] Pan ZF Zhou Y Ruan Y Luo X Yan YJ Zhou L. Catechins attenuate inflammatory responses in a murine model of allergic asthma by inhibiting the NF-κB–TSLP signaling pathway. Chin Pharmacol Bull. (2018) 34:207–12.

[51] Qi HJ Li YL Wu JX Xie SQ Li HJ Wang XP. Elevated expression of TSLP in airway epithelium aggravates airway inflammation in patients with asthma. J Shandong Univ (Health Sciences). (2011) 49:143–8.

[52] Portacci A Scioscia G Dragonieri S Aliani M Lulaj E Montagnolo F. The impact of Tezepelumab therapy on perceived asthma triggers: a multicentre real-life study. J Asthma. (2025) 21:1–12. doi: 10.1080/02770903.2025.2495725, PMID: 40257396

[53] Inoue S Ogata H Dotake Y Takagi K Shiota A Ishii Y. Tezepelumab achieves improvement of severe uncontrolled asthma and rhinosinusitis: Case series. J Allergy Clin Immunol Glob. (2025) 4:100448. doi: 10.1016/j.jacig.2025.100448, PMID: 40226773 PMC11986613

[54] Lamme TD Smit MJ Schafer CT. Signal termination of the chemokine receptor CCR9 is governed by an arrestin-independent phosphorylation mechanism. J Biol Chem. (2025) 301:108462. doi: 10.1016/j.jbc.2025.108462, PMID: 40154615 PMC12147180

[55] Li N Wang H Zhao H Wang M Cai J Hao Y. Cooperative interactions between Veillonella ratti and Lactobacillus acidophilus ameliorate DSS-induced ulcerative colitis in mice. Food Funct. (2023) 14:10475–92. doi: 10.1039/D3FO03898J, PMID: 37934670

[56] Korsten SGPJ Vromans H Garssen J Willemsen LEM. Butyrate protects barrier integrity and suppresses immune activation in a caco-2/PBMC co-culture model while HDAC inhibition mimics butyrate in restoring cytokine-induced barrier disruption. Nutrients. (2023) 15:2760. doi: 10.3390/nu15122760, PMID: 37375664 PMC10305054

[57] Zhang Y Han J Gao J Ge Q Zhang H Shi J. Polysaccharide from Pyrus pashia Buch ameliorates DSS-induced colitis in mice via MAPKP38/NF-κB P65 and SCFAs/ERK/MSK signaling pathways. Phytomedicine. (2025) 140:156561. doi: 10.1016/j.phymed.2025.156561, PMID: 40036991

[58] Fan X Zhang Z Gao W Pan Q Luo K He B. An engineered butyrate-derived polymer nanoplatform as a mucosa-healing enhancer potentiates the therapeutic effect of magnolol in inflammatory bowel disease. ACS Nano. (2024) 18:229–44. doi: 10.1021/acsnano.3c05732, PMID: 38112525

[59] Gong X Geng H Yang Y Zhang S He Z Fan Y. Metabolic engineering of commensal bacteria for gut butyrate delivery and dissection of host-microbe interaction. Metab Eng. (2023) 80:94–106. doi: 10.1016/j.ymben.2023.09.008, PMID: 37717646

[60] Mao ML Lin P Xiong LL Feng Y Shu QL. Effects of Shenling Baizhu Powder and Lizhong Decoction on the diversity of butyrate-producing gut microbiota in an antibiotic-associated diarrhea animal model. Chin J Exp Traditional Med Formulae. (2021) 27:23–30. doi: 10.13422/j.cnki.syfjx.20212106

[61] Lin P Qiu C Shu QL Feng Y. Microecological mechanism of *in vitro* degradation of Shenling Baizhu Powder polysaccharides based on a consortium of butyrate-producing bacteria. China J Traditional Chin Med Pharm. (2023) 38:620–5.

